# Changes in Dietary Fat Content Rapidly Alters the Mouse Plasma Coagulation Profile without Affecting Relative Transcript Levels of Coagulation Factors

**DOI:** 10.1371/journal.pone.0131859

**Published:** 2015-07-15

**Authors:** Audrey C. A. Cleuren, Vicky T. Blankevoort, Janna A. van Diepen, Daniël Verhoef, Peter J. Voshol, Pieter H. Reitsma, Bart J. M. van Vlijmen

**Affiliations:** 1 Einthoven Laboratory for Experimental Vascular Medicine, Department of Thrombosis and Hemostasis, Leiden University Medical Center, Leiden, the Netherlands; 2 Einthoven Laboratory for Experimental Vascular Medicine, Department of Endocrinology, Leiden University Medical Center, Leiden, the Netherlands; INRA, FRANCE

## Abstract

**Background:**

Obesity is associated with a hypercoagulable state and increased risk for thrombotic cardiovascular events.

**Objective:**

Establish the onset and reversibility of the hypercoagulable state during the development and regression of nutritionally-induced obesity in mice, and its relation to transcriptional changes and clearance rates of coagulation factors as well as its relation to changes in metabolic and inflammatory parameters.

**Methods:**

Male C57BL/6J mice were fed a low fat (10% kcal as fat; LFD) or high fat diet (45% kcal as fat; HFD) for 2, 4, 8 or 16 weeks. To study the effects of weight loss, mice were fed the HFD for 16 weeks and switched to the LFD for 1, 2 or 4 weeks. For each time point analyses of plasma and hepatic mRNA levels of coagulation factors were performed after overnight fasting, as well as measurements of circulating metabolic and inflammatory parameters. Furthermore, in vivo clearance rates of human factor (F) VII, FVIII and FIX proteins were determined after 2 weeks of HFD-feeding.

**Results:**

HFD feeding gradually increased the body and liver weight, which was accompanied by a significant increase in plasma glucose levels from 8 weeks onwards, while insulin levels were affected after 16 weeks. Besides a transient rise in cytokine levels at 2 weeks after starting the HFD, no significant effect on inflammation markers was present. Increased plasma levels of fibrinogen, FII, FVII, FVIII, FIX, FXI and FXII were observed in mice on a HFD for 2 weeks, which in general persisted throughout the 16 weeks of HFD-feeding. Interestingly, with the exception of FXI the effects on plasma coagulation levels were not paralleled by changes in relative transcript levels in the liver, nor by decreased clearance rates. Switching from HFD to LFD reversed the HFD-induced procoagulant shift in plasma, again not coinciding with transcriptional modulation.

**Conclusions:**

Changes in dietary fat content rapidly alter the mouse plasma coagulation profile, thereby preceding plasma metabolic changes, which cannot be explained by changes in relative expression of coagulation factors or decreased clearance rates.

## Introduction

The prevalence of obesity in the Western world is rising, which causes a public health problem since obesity affects, amongst others, the development of cardiovascular diseases through its influence on risk factors like hyperlipidemia, hypertension, glucose intolerance and inflammation. The risk for thrombotic cardiovascular events is even further enhanced by the hypercoagulable state that is associated with obesity, as obese subjects have increased plasma levels of procoagulant factor (F) VII, VIII, XII and fibrinogen, while fibrinolysis is decreased as reflected by increased levels of plasminogen activator inhibitor-1 (PAI-1) [1–3]. On the other hand, levels of the anticoagulant factors protein C and protein S are higher, whereas tissue plasminogen activator (tPA) levels are lower under obese conditions, which might be considered to be a compensatory response to the hypercoagulable state [4,5].

Previous studies evaluating the effect of weight loss on hemostatic parameters showed that levels of tissue factor, FVII, and PAI-1 decreased upon weight loss, resulting in a decrease in thrombin generation [6,7]. In addition, it has been suggested that almost one-third of all thrombotic events could be prevented by weight loss [8]. Taken together, these data indicate that plasma coagulation factors, and the subsequent thrombotic risk, may follow both the unfavorable and favorable changes in body weight gain and loss, respectively.

Using an experimental animal approach, we and others have previously shown that obesity in mice also results in a hypercoagulable state, which is characterized by increased plasma levels of procoagulant factors and decreased fibrinolysis [9,10]. These results were obtained in mice that had been on a high fat diet for 4 to 5 months, and during this time many other metabolic changes may have occurred influencing the coagulation profile indirectly. Therefore, we now establish the onset and reversibility of the hypercoagulable state during the development and regression of nutritionaly-induced obesity by combining high fat and subsequent low fat feeding in a mouse model. To further evaluate the potential mechanism leading to the changes in the plasma coagulation profile, we also determine its relation to transcriptional changes and clearance rates of a subset of coagulation factors.

## Materials and Methods

### Animals

Six week old male C57BL/6J mice (Charles River, Maastricht, the Netherlands) were fed a diet with 10% kcal as fat content (low fat diet, D12450B; Research Diets, New Brunswick, NJ) for 4 weeks as a run-in period, after which half of the group switched to an iso-caloric diet with 45% kcal as fat content (high fat diet, D12451; Research Diets), while the other group remained on the low fat diet (LFD). After 2, 4, 8 or 16 weeks mice (n = 15 per group) were fasted overnight and subsequently anesthetized with a mixture of ketamine, xylazine and atropine. The abdomen was opened and a blood sample on sodium citrate (final concentration of 0.32%) was directly drawn from the inferior caval vein. Platelet-poor plasma was obtained and stored at -80°C until use, and part of the left liver lobule was snap-frozen for mRNA analyses.

In order to compare nutritionally-induced obesity with genetically-induced obesity, 6 week old *ob/ob* mice, and their lean wild-type littermate controls (Charles River) were fed the LFD for 4 weeks and plasma and tissue samples were obtained for analyses after overnight fasting.

Plasma clearance of the vitamin K-dependent coagulation factors VIII, VII and IX were determined in a separate experiment in which mice fed a HFD for 2 weeks received a single intravenous injection (200 μl) of either the human FVIII concentrate (Aafact, Sanquin Plasma Products, Amsterdam, the Netherlands) or human prothrombinase complex concentrate (Cofact, Sanquin Plasma Products, Amsterdam, the Netherlands; both kindly provided by Dr. K. Mertens, Sanquin). Clearance rates of the human plasma-derived factors from the individual mouse plasmas were determined by successive blood sampling in EDTA vials via the tail vein.

To study the effects of weight loss after nutritionally-induced obesity, mice receiving the HFD for 16 weeks were switched to the LFD (n = 45), while part remained on the HFD (n = 45). After 1, 2 or 4 weeks, mice were sacrificed after overnight fasting for plasma and tissue analyses.

All experimental animal procedures were approved by the animal welfare committee of the Leiden University.

### Plasma analyses

Plasma triglyceride and insulin levels were measured using commercially available kits (Roche Molecular Biochemicals, Indianapolis, IN, and Crystal Chem Inc., Downers Grove, IL, USA) and glucose levels were determined according to the hexokinase method (Instruchemie). Plasma levels of multiple cytokines were evaluated simultaneously by using pre-coated multisport plates in an ELISA-based electrochemiluminescence assay (Meso Scale Discovery, Gaithersburg, MD).

Coagulation factor levels were measured as previously described [11] and pooled mouse plasma was used to generate standard curves. Global coagulability of the plasma was determined by measuring the prothrombin time (PT) and activated partial thromboplastin time (aPTT) on the STart 4 analyzer (Diagnostica Stago, Leiden, The Netherlands) using the STA Neoplastine Plus (Diagnostica Stago) and the TriniCLOT Automated APTT reagent (TCoag, Ireland), respectively.

The *in vivo* clearance rates of human coagulation factors VII, VIII and IX were analyzed with home-made ELISAs specific for human proteins which did not cross-react with mouse plasma proteins. Standard curves were generated by adding Cofact or Aafact to pooled mouse plasma (final concentration 20%) to calculate human antigen levels, and the level measured directly after injection (1 minute) was set as a reference (100%).

### RNA isolation and real-time RT-PCR

Individual liver samples (15–20 mg) of 10 animals per group were homogenized in RNAzol (Bio-Connect, Huissen, the Netherlands), and RNA isolation and cDNA synthesis was executed as previously reported [11]. Quantitative real-time PCR was performed using SybrGreen (Life Technologies, Bleiswijk, the Netherlands) and gene-specific primers [11]. The comparative threshold cycle method with ß-actin as internal control was used for quantification and normalization. To evaluate the effects of weight gain on transcript levels, LFD-fed mice were set as a reference, whereas the HFD-fed mice were set as a reference to determine the effects of weight loss. The ΔC_t_ values of individual samples were related to the mean ΔC_t_ of the reference group.

### Statistical analyses

Normally distributed data are expressed as mean ± standard error of the mean (SEM) and are evaluated using a Student’s t-test. Data that does not follow a normal distribution, i.e. inflammatory markers, are presented as the median including the range and are analyzed using a Mann-Whitney test. Gene expression data are presented as mean together with the minimum and maximum expression levels. Data analyses were performed with the GraphPad Instat software (San Diego, CA) and differences between the LFD and HFD groups were considered statistically significant at a p-value <0.05, which after the Bonferroni correction for multiple testing equals an adjusted p-value <0.0025.

## Results

### Induction of obesity

Two-week HFD-feeding resulted in a significantly increased fasted body weight as compared to the LFD-fed mice (25.0±0.6 g vs. 22.3±0.3 g, p<0.001), which gradually increased further over time (Table 1, S1 Fig). From 8 week onwards, liver weights of HFD-fed mice were also significantly higher than those of LFD-fed mice (at 8 weeks, 0.77±0.01 g vs. 0.95±0.02 g, p<0.001, S2 Fig). Triglycerides and insulin levels were increased after 16 week high fat diet, whereas plasma glucose values were significantly increased at 8 weeks with 5.7±0.2 mmol/L for LFD and 7.7±0.4 mmol/L for HFD (p<0.001; Table 1, S3–S5 Figs).

**Table 1:** Metabolic parameters of mice on a low fat diet (LFD) or high fat diet (HFD) for 16 weeks as compared to genetically obese *ob/ob* mice with their littermate wild-type controls after 4 weeks of LFD feeding.

	LFD (n = 15)	HFD (n = 15)	WT (n = 15)	*ob/ob* (n = 15)
Body weight (g)	27.4±0.5	41.6±0.9^‡^	22.1±0.6	40.3±0.6^‡^
Liver weight (g)	0.79±0.01	1.08±0.06^‡^	0.78±0.04	2.14±0.06^‡^
Triglycerides (mmol/L)	0.54±0.03	0.65±0.04*	0.62±0.05	0.39±0.02^‡^
Insulin (pg/mL)	97.4±1.0	105.0±2.8*	94.1±0.9	105.4±1.8^‡^
Glucose (mmol/L)	5.7±0.2	8.0±0.6^‡^	5.8±0.6	11.6±0.9^‡^

Data are expressed as mean±SEM.

*p<0.05 and

^‡^p<0.001 as compared to LFD-fed mice or wild-type controls as appropriate.

Plasma cytokine levels showed a transient rise in interleukin (IL) 1ß, IL-6, IL-12 and keratinocyte chemoattractant (KC) levels after 2 weeks of HFD-feeding (2.7 (0.7–20.7) pg/mL vs. 6.0 (1.1–88.9) pg/mL for IL-1β, p<0.05; 113.5 (33.5–2318.2) pg/mL vs. 398.3 (86.0–2282.2) pg/mL for IL-6, p<0.05; 82.0 (35.2–2886.7) pg/mL vs. 247.7 (112.7–2166.6) pg/mL for IL-12, p<0.05; 34.6 (19.1–246.8) pg/mL vs. 55.9 (28.7–233.6) pg/mL for KC, p<0.05). The levels of IL-10, interferon-γ (IFN-γ) and tumor necrosis factor α (TNFα) were not affected (data not shown).

The high fat feeding-related changes in metabolic parameters after 16 weeks largely resembled the observations in the LFD-fed *ob/ob* mice, which had a comparable body weight and plasma insulin levels (Table 1, S1–S5 Figs). As compared to the diet-induced obese mice, the *ob/ob* mice had more pronounced increases in liver weight and glucose levels, although they displayed a surprisingly lower fasted plasma triglyceride level (Table 1). KC and IFN-γ levels were higher in *ob/ob* mice (KC 40.4 (16.5–229.1) pg/mL vs. 100.1 (17.6–268.9) pg/mL, p<0.05; IFN-γ 7.3 (2.0–53.9) pg/mL vs. 45.9 (1.2–125.0) pg/mL, p<0.05) than in their wild-type littermate controls.

**Fig 1:**
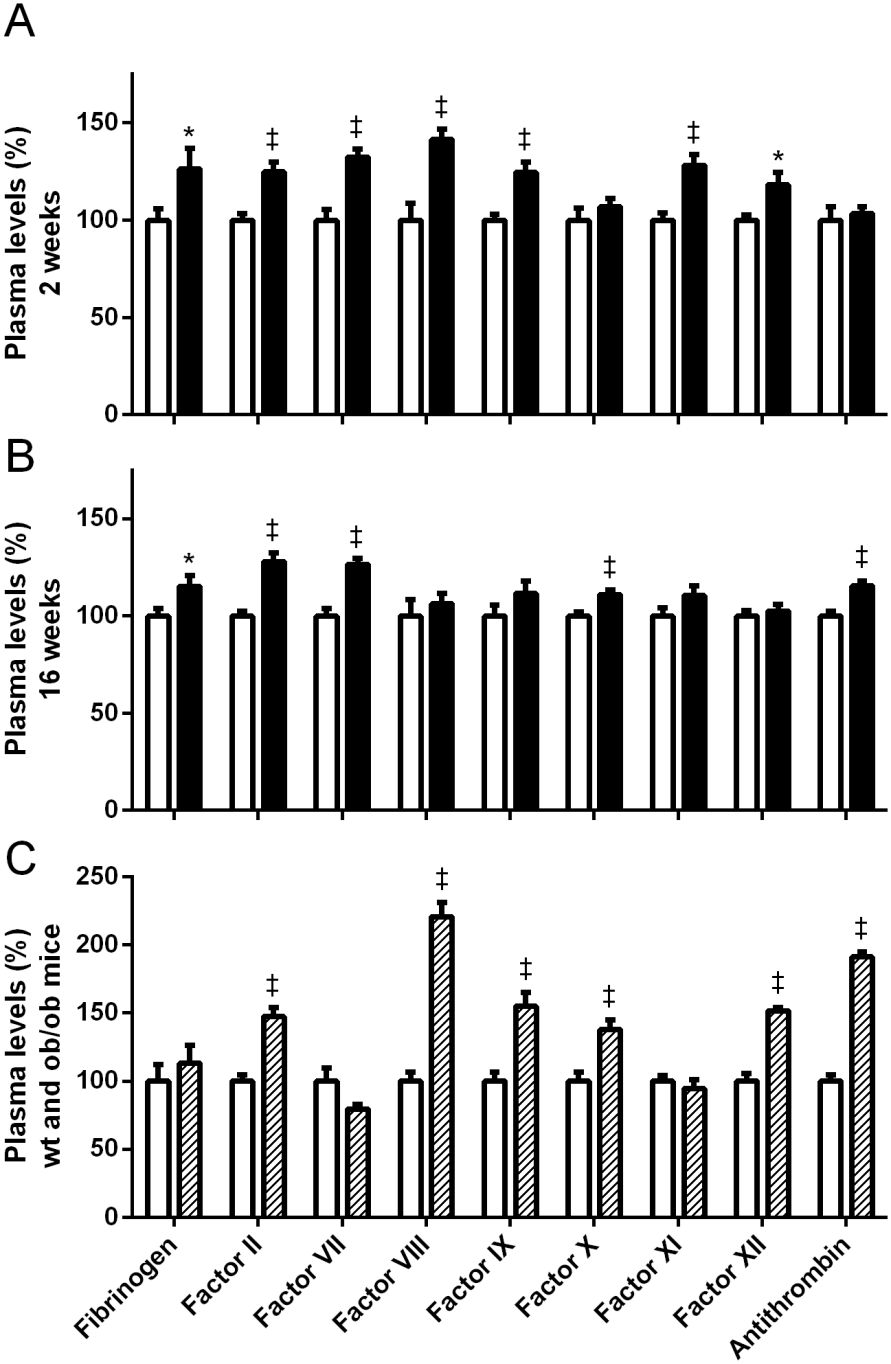
Effects low fat diet (LFD) and high fat diet (HFD) on plasma coagulation parameters. Effects on plasma coagulation parameters after 2 (panel A) and 16 (panel B) weeks of low fat diet (white) or high fat diet (black) feeding. Panel C shows the plasma coagulation profile of genetically obese *ob/ob* mice (striped) and their wild-type littermates (white) after 4 weeks on a low fat diet. Data are presented as mean±SEM. *p<0.05 and ^‡^p<0.001 as compared to the LFD-fed mice or wild-type controls as appropriate.

Already within 2 weeks of high fat feeding, a clear procoagulant shift of the plasma coagulation profile was observed with significant increases in FII, FVII, FVIII, FIX and FXI (p<0.001) and higher FXII and fibrinogen levels (p<0.05) while FX and antithrombin levels remained unaffected (Fig 1A). Continuation of the HFD resulted in sustained increased levels of fibrinogen, FII and FVII whereas the effects on plasma FVIII, FIX, FXI and FXII levels disappeared after 16 weeks HFD-feeding, and FX and antithrombin levels were only significantly higher after 16 weeks of HFD-feeding (Fig 1B). These effects on the individual coagulation factor activity levels were also reflected in the overall coagulability of the plasma as the HFD-fed mice had shorter times to clot in the PT and aPTT assays, indicating a procoagulant shift of the plasma coagulation profile (2 weeks: PT 13.8±0.3 vs 14.5±0.1 s, p<0.05, aPTT 33.9±0.5 vs 40.3±1.2 s, p<0.001; 16 weeks: PT 13.6±0.1 vs 14.1±0.1 s, p<0.05, aPTT 37.1±0.6 vs 42.1±0.7 s, p<0.001). Compared to the 16 week HFD-fed animals, the *ob/ob* mice showed a similar procoagulant shift in individual coagulation factors which were again more prominent (Fig 1C). Remarkably, factor VII levels, similar to the triglycerides, were lower in *ob/ob* mice than in the wild-type controls and plasma levels of fibrinogen and FXI were not significantly affected. Like the 16 week HFD-fed animals, the *ob/ob* mice also showed shorter clotting times in the overall PT and aPTT analyses (PT 14.1±0.1 vs 16.1±0.5 s, p<0.001, aPTT 31.5±0.7 vs 34.6±0.7 s, p<0.01).

Since the liver is the main site of production of plasma coagulation factors, we determined whether the changes in the plasma coagulation profile due to the high fat diet were related to changes in hepatic transcript levels, as we have previously shown that changes in plasma levels can coincide with transcriptional effects [11]. Although at the 2-week time point the liver weight between diet treatment groups are comparable (0.72±0.02 g for LFD and 0.74±0.01 g for HFD), and a clear increase in plasma levels of coagulation factors was observed, relative mRNA levels of coagulation genes were not affected, with the exception of *F11* which was significantly increased (Table 2). Surprisingly, whereas the FVIII plasma levels were significantly increased, its transcript levels were significantly lower following HFD feeding (Fig 1A). Despite the differences in liver weight after 16 weeks of high fat-feeding, we evaluated whether prolonged exposure to dietary fat was able to affect transcription. However, besides the significant decrease in *F12* mRNA (LFD: 1 (0.93–1.07), HFD: 0.72 (0.69–0.76); p = 0.001), long-term HFD-feeding also did not result in significant changes in relative mRNA levels of hepatically expressed coagulation factors (S1 Table).

**Table 2:** Hepatic mRNA levels of coagulation genes of mice on a low fat diet (LFD) or high fat diet (HFD) for 2 weeks.

	LFD (n = 10)	HFD (n = 10)
Fibrinogen	1 (0.93–1.08)	0.87 (0.83–0.90)
Factor II	1 (0.93–1.08)	1.05 (0.97–1.13)
Factor VII	1 (0.95–1.05)	1.07 (1.01–1.13)
Factor VIII	1 (0.94–1.06)	0.64 (0.54–0.77)*
Factor IX	1 (0.95–1.05)	1.08 (1.03–1.13)
Factor X	1 (0.96–1.04)	1.01 (0.96–1.07)
Factor XI	1 (0.92–1.09)	1.51 (1.44–1.58)^‡^
Factor XII	1 (0.95–1.05)	1.06 (1.01–1.12)
Antithrombin	1 (0.95–1.05)	1.05 (1.00–1.10)

Data are expressed as mean (minimum-maximum expression level).

*p<0.05 and

^‡^p<0.001 as compared to LFD mice.

**Fig 2:**
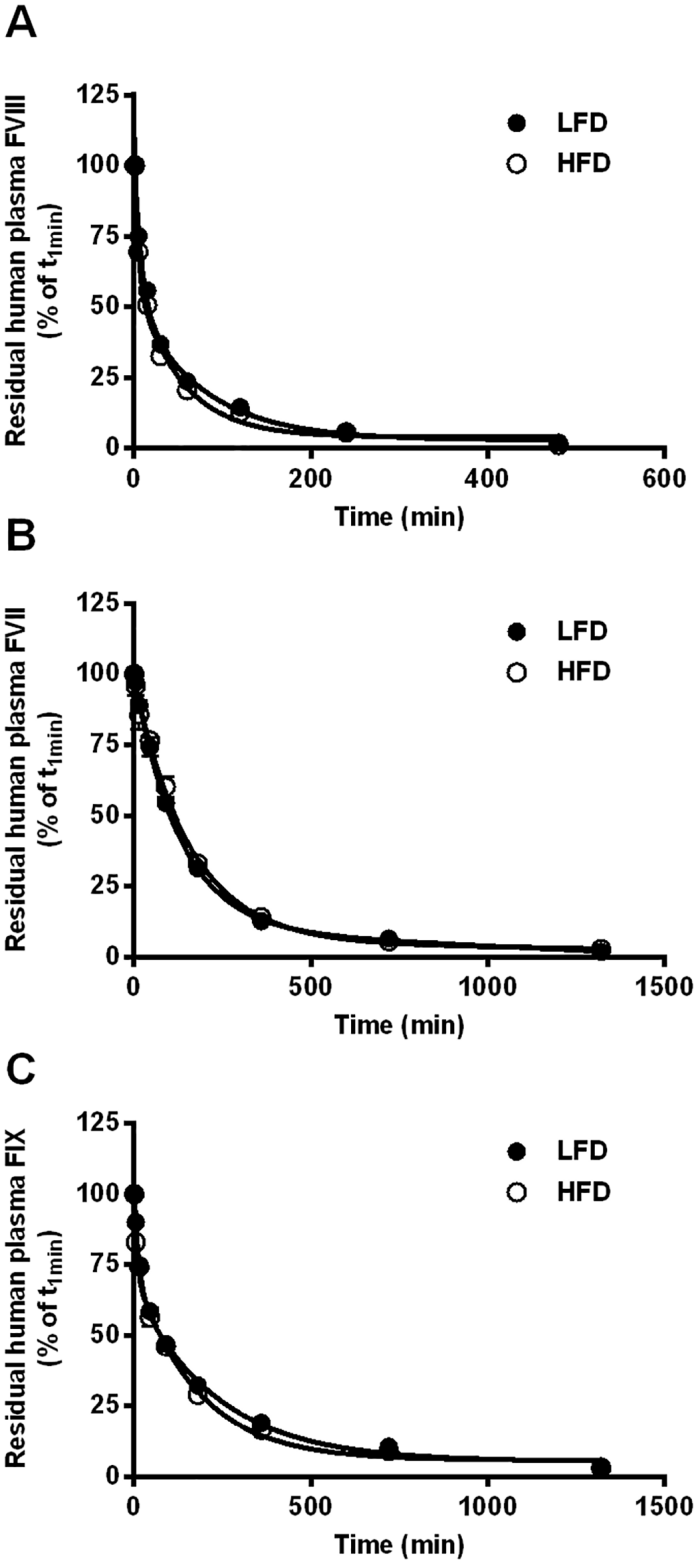
Effect of low fat diet (LFD) and high fat diet (HFD) on plasma clearance of FVIII and the vitamin K-dependent coagulation factors VII and IX. Plasma clearance of FVIII (panel A) and the vitamin K-dependent coagulation factors VII (panel B) and IX (panel C) were determined in a separate experiment in which mice that were on a LFD (closed symbols) or HFD (open symbols) for 2 weeks received a single intravenous injection (200 μl) of either human FVIII concentrate (Aafact) or the human prothrombinase complex concentrate (Cofact). Clearance rates of the human plasma-derived factors from the mouse plasma were determined by successive blood sampling in EDTA vials via the tail vein and factor levels determination by ELISA. Values are the mean±SEM of six mice and expressed as the percentage of factor remaining in the circulation, with the amount of factor present at 1 minute after injection considered as 100%. Curves were calculated from the mean data using a one-exponential curve fit model.

As the changes in the plasma coagulation profile were not paralleled by changes in relative expression levels, we determined whether HFD-feeding for 2 weeks affected plasma protein turnover by decreasing clearance rate. A bolus injection of either the human FVIII concentrate or human prothrombin complex concentrate resulted in both the HFD and LFD group in single-phase clearance curves with comparable half-lives between LFD-fed and HFD-fed mice (FVIII 18.6±1.8 min vs. 15.3±1.7 min, FVII 112.6±7.1 min vs. 99.4±5.9 min and FIX 79.0±9.5 vs. 76.5±5.7 min; Fig 2).

### Regression of obesity

Since the dietary fat intake resulted in a rapid procoagulant shift of the plasma coagulation profile, we determined whether regression of the nutritionally-induced obesity also altered coagulation. Therefore, part of the mice receiving the HFD for 16 weeks switched to the LFD (n = 45) while the remaining mice continued on the HFD (n = 45). Switching to the LFD resulted in a decrease in body weight within a week (37.3±1.5 g vs. 42.9±1.1 g, p<0.01, S1 Fig), while effects on the liver weight were apparent after 2 weeks (1.05±0.06 g vs. 1.22±0.08 g, p<0.05, S2 Fig). Fasted plasma glucose levels were also rapidly affected (6.4±0.4 mmol/L vs. 8.7±0.7 mmol/L, p<0.01, S5 Fig), whereas insulin levels went down after 4 weeks being switched to the LFD (103.5±1.0 pg/mL for LFD switch vs. 109.3±2.4 pg/mL for the HFD mice, p<0.05, S4 Fig) and triglyceride levels remained comparable between the 2 groups (S3 Fig). With the exception of a transient increase in KC levels (58.2 (36.1–168.2) pg/mL for HFD vs. 90.8 (36.3–542.6) pg/mL after switch, p<0.05), none of the inflammatory cytokines levels were affected as a result of the diet switch.

**Fig 3:**
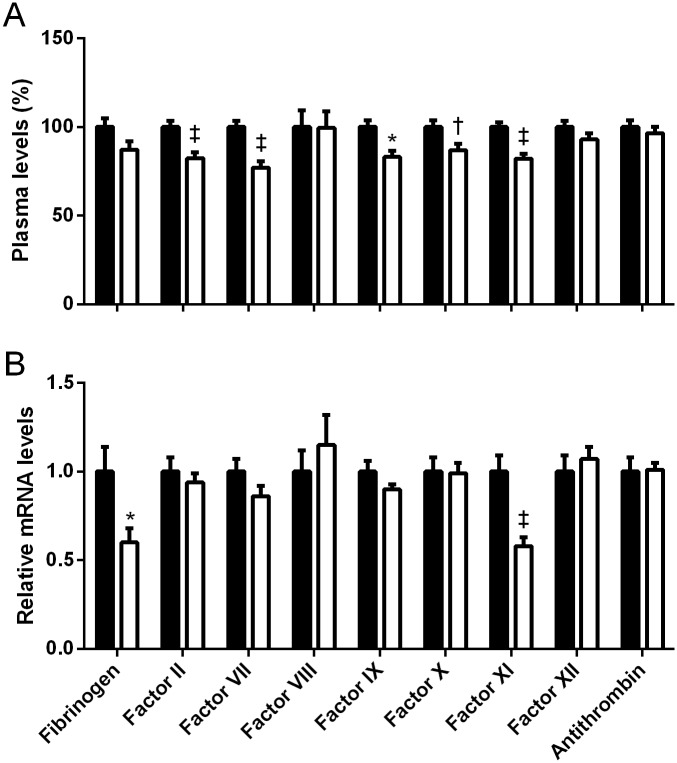
Effects on plasma and relative transcript levels of mice on a high fat diet (HFD) for 17 weeks and mice that were switched after 16 weeks of HFD-feeding to the low fat diet (LFD) for 1 week. Plasma (A) and relative transcript levels (B) of mice on a high fat diet for 17 weeks (black) and mice that were switched after 16 weeks of HFD-feeding to the LFD for 1 week (white). Data are presented as mean±SEM for the plasma data and as mean with the error bar representing the calculated maximum expression level of n = 10 mice per group for the expression levels. Relative expression levels were compared using the comparative threshold cycle method with ß-actin as internal control and the HFD-fed mice were set as a reference. *p<0.05, †p<0.01 and ‡p<0.001 as compared to the LFD-fed mice or wild-type controls as appropriate.

The plasma coagulation profile showed a remarkably rapid shift after switching to a LFD, with significantly reduced activity levels of FII, FVII and FXI, and additional decreased levels of FIX and FX after only 1 week (Fig 3A), which all persisted throughout the remaining for 4 weeks after switching to the LFD. The initial changes in individual factors after 1 week resulted a prolonging of the PT (13.1±0.1 vs 14.1±0.4 s, p<0.05), while the aPTT was not affected (38.5±0.7 vs 38.4±1.0 s). In addition, factor VIII and antithrombin levels were altered after 2 weeks of switching diets (100±9.8% for mice remaining on the HFD vs. 72.4±5.0%, p<0.01 and 100±2.3% vs. 86.6±4.4, p<0.05 respectively), whereas FXII levels were lower after 4 weeks (100±1.5% vs. 92.1±1.5, p<0.01). As one week after switching diets was able to induce alterations in the plasma coagulation profile, while liver weights were not significantly affected (1.22±0.08 g for HFD vs 1.05±0.06 g for HFD switched to LFD), hepatic mRNA analyses were performed to determine whether relative transcript levels of coagulation genes were modulated by the diet switch. While similar trends between plasma and hepatic transcript levels were observed for several coagulation factors, the significant decrease in plasma FXI activity was the only value paralleled by a significant reduction in transcript level (Fig 3).

## Discussion

Obesity is an increasing public problem and is associated with numerous health issues, including a hypercoagulable state. In order to gain more insight in the relation between obesity and hypercoagulability we used an *in vivo* approach to study the onset and potential reversibility of the hypercoagulable state during the development and regression of nutritionally-induced obesity. In addition, we determined the mechanisms leading to this hypercoagulable state by evaluating transcription and clearance of several coagulation factors, as well as determining metabolic and inflammatory parameters since they may aggravate the hypercoagulable state associated with obesity.

We have shown that nutritionally-induced obesity coincides with an early-onset procoagulant shift of the plasma coagulation profile, which was already apparent within 2 weeks after the start of the HFD. This appeared to be independent of changes in relative transcript levels and clearance rates of coagulation factors as measured for a limited subset. Furthermore, these changes in the plasma coagulation profile largely persisted during the continuation of the HFD for 16 weeks, thereby preceding the effects in metabolic parameters like glucose and insulin levels since these were only affected in overt obese mice. Switching from a high fat to a low fat diet to induce weight loss resulted in a rapid reversal of the HFD-induced procoagulant shift of the plasma coagulation profile, as observed within 1 week after switching diets.

A remarkable finding in this study is that the changes present in plasma activity levels after 2 weeks of HFD feeding could not be explained by changes in relative transcript levels of hepatically expressed coagulation factors, nor by changes in clearance rates as measured for several individual factors. This difference between mRNA levels and clearance rates on the one hand, and plasma levels on the other, may have several reasons. First of all, human proteins were used to study potential changes in clearance, but these may be differently cleared than the endogenous murine proteins. The use of mouse recombinant or plasma derived proteins would have been more physiologic to study clearance. However, purified mouse proteins were not available and the instability of these proteins ex vivo preclude isolation and (radio)labeling for autologous injections and thus the use in plasma clearance studies. Regarding the mRNA levels, high fat feeding may affect liver physiology. Therefore we were interested whether the RNA recovery (μg RNA/mg liver) in liver samples of LFD and HFD mice differed. Although the liver weights after 2 weeks of HFD feeding were comparable, the total amount of RNA recovered per mg liver weight of HFD mice was approximately 20% higher as compared to that of the LFD-fed mice. One can speculate that although the relative levels compared to ß-actin remain similar, the overall increase in total mRNA may result in an absolute increase in transcript levels and therefore still contribute to the increased plasma activity levels. In addition, post-transcriptional or post-translational mechanisms may affect the activity of the resulting protein as well. For example, phosphoenolpyruvate carboxykinase (PEPCK), an enzyme associated with hepatic glycogen storage, is transcriptionally down-regulated under nutritionally-induced obesity while the protein activity is increased [12]. Finally, for several coagulation factors it is known that they are influenced by circadian rhythms [13]. In order to prevent a potential bias, for each time point LFD and HFD mice were sacrificed for blood and liver isolation within the same time frame in an alternating manner. Nevertheless, we cannot exclude a potential (circadian driven) discrepancy between transcript levels and actual protein activity levels in the plasma. Although we were not able to pinpoint the exact mechanisms by which the effects on the plasma coagulation profile under nutritionally-induced obesity and regression occur, we were able to show that these results happen independently of metabolic and inflammatory changes.

Another remarkable observation was the contradicting result on FVIII, where the plasma FVIII increased while the relative mRNA levels decreased. In this respect it is interesting that FVIII, unlike the majority of the circulating coagulation factors, is produced not by hepatocytes but by endothelial cells [14,15]. In addition, it was shown that the liver is not the only site of FVIII production as the endothelial cells in the kidney are also capable of generating FVIII [14]. Although at this point we can only speculate on the potential differences in FVIII production at other sites than the liver; it might be possible that FVIII under HFD conditions is differentially expressed (i.e. increased) in endothelial cells in other organs as a compensatory mechanism for the reduction observed in the liver, thereby causing an increased FVIII activity in plasma.

As shown in Fig 1A and 1B the coagulation factors seem to be categorized into 2 distinct groups based on their changes in the plasma levels after 2 and 16 weeks of HFD. The first category, consisting of fibrinogen, FII and FVIII are increased after 2 weeks of HFD feeding and remain increased throughout the 16 week diet. These are all associated with obesity and insulin resistance or elevated triglycerides (2), which make them likely targets to remain increased throughout the induction of obesity and the accompanying metabolic changes. On the other hand, the intrinsic coagulation factors VIII, IX, XI and XII only show a transient increase at 2 weeks. The elevation in FVIII might be explained by its role as acute phase protein and is in line with the temporary rises seen in cytokine levels. However, the associations between these intrinsic factors and obesity seem to be less defined, potentially explaining the lack of responses after established obesity on our mouse model.

The transient rise in plasma cytokine levels when mice switch to the HFD suggests that the system has to adapt to the new diet in order to maintain homeostasis. These data are in concordance with the metabolic stress response that occurs during short-term high fat feeding [16]. Furthermore, a previous genome-wide mRNA expression study which focused on changes in hepatic gene expression during high fat feeding, also showed that exposure to dietary fat first results in inflammation which under long-term high fat feeding causes a switch to a steatotic transcriptional program [17].

Besides nutritionally-induced obesity, we included genetically obese *ob/ob* mice and their wild-type littermate controls in our experiments, mainly as a control for determining the effects of HFD on metabolic parameters like insulin resistance. The *ob/ob* mice have been predominantly used to study metabolic disorders leading to type 2 diabetes, and although they have been used in studies focusing on tissue factor and PAI-1 [18,19] in general little is known about their overall plasma coagulation profile. Here we show that *ob/ob* mice have more pronounced increases in plasma procoagulant factor levels as compared to diet-induced obese mice that have been on a HFD for 16 weeks, with the exception of FVII levels. These metabolic abnormalities in *ob/ob* mice may aggravate the hypercoagulable state, for example via an increased transcriptional activity of nuclear factor (NF)-κB which can induce expression of coagulation genes. Because of the underlying pathologies that can potentially affect coagulation, the *ob/ob* mouse seems to be less suitable to study obesity with respect to coagulation and a nutritionally-induced obesity model seems warranted.

By studying coagulation during the development and regression of nutritionally-induced obesity, we were able to show that the dietary fat content plays and important role in affecting the plasma coagulation profile. It was recently shown that the endogenous thrombin potential (ETP) was increased in rats on a high fat diet, and the PT and aPTT shortened in diet-induced obese mice [20,21]. This is in line with our data showing a hypercoagulable state in mice on a HFD based on individual coagulation factors as well as the functional measurements of coagulation by PT and aPTT analyses. Although the study by Sanchez et al. did not observe a rapid decrease in ETP after switching the rats back to a low fat diet but instead saw an immediate positive effect on insulin levels, these studies show that dietary fat and obesity are important modulators of the coagulation profile. Thus, dietary intervention could improve the coagulation profile and therefore be beneficial in the primary prevention of thrombosis.

In summary, this *in vivo* study shows that the plasma coagulation profile is able to rapidly respond to changes in dietary fat content, as an increase in weight is associated with a procoagulant shift, whereas subsequent weight loss results in a reversal of the HFD-induced hypercoagulability. These changes in the plasma coagulation profile appear to be independent of changes in relative transcript levels of coagulation genes and changes in clearance as evaluated by measuring *in vivo* clearance rates of human proteins. In addition, the effects on coagulation precede alterations in metabolic parameters like insulin and glucose levels. The fact that weight loss is associated with rapid beneficial effects on coagulation may eventually translate in a risk reduction for thrombotic cardiovascular events.

## Supporting Information

S1 Table Hepatic mRNA levels of coagulation genes of mice on a low fat diet (LFD) or high fat diet (HFD) for 16 weeks. (DOCX)

S1 Fig Body weight of mice. Left panel, left of vertical solid line: Body weight of mice on a low fat diet (LFD, open symbols) or high fat diet (HFD, filled symbols) for 0, 2, 4, 8, 16 weeks. Left panel, right of vertical solid line: Body weight of mice on a high fat diet for 17, 18, 19 or 20 weeks (HFD, filled symbols) and mice that were switched after 16 weeks of HFD-feeding to the LFD for 1, 2, or 4 weeks (LFD, open symbols). Right panel: Body weight of genetically obese *ob/ob* mice (filled symbols) with their littermate wild-type controls (open symbols) after 4 weeks of LFD feeding. Horizontal line indicates the mean for the body weight data.
(TIF)

S2 Fig Liver weight of mice. Left panel, left of vertical solid line: Liver weight of mice on a low fat diet (LFD, open symbols) or high fat diet (HFD, filled symbols) for 0, 2, 4, 8, 16 weeks. Left panel, right of vertical solid line: Liver weight of mice on a high fat diet for 17, 18, 19 or 20 weeks (HFD, filled symbols) and mice that were switched after 16 weeks of HFD-feeding to the LFD for 1, 2, or 4 weeks (LFD, open symbols). Right panel: Liver weight of genetically obese *ob/ob* mice (filled symbols) with their littermate wild-type controls (open symbols) after 4 weeks of LFD feeding. Horizontal line indicates the mean for the liver weight data.
(TIF)

S3 Fig Plasma triglyceride levels of mice. Left panel, left of vertical solid line: plasma triglyceride levels of mice on a low fat diet (LFD, open symbols) or high fat diet (HFD, filled symbols) for 0, 2, 4, 8, 16 weeks. Left panel, right of vertical solid line: plasma triglyceride levels of mice on a high fat diet for 17, 18, 19 or 20 weeks (HFD, filled symbols) and mice that were switched after 16 weeks of HFD-feeding to the LFD for 1, 2, or 4 weeks (LFD, open symbols). Right panel: plasma triglyceride levels of genetically obese *ob/ob* mice (filled symbols) with their littermate wild-type controls (open symbols) after 4 weeks of LFD feeding. Horizontal line indicates the mean for the plasma triglyceride levels.
(TIF)

S4 Fig Plasma insulin levels of mice. Left panel, left of vertical solid line: plasma insulin levels of mice on a low fat diet (LFD, open symbols) or high fat diet (HFD, filled symbols) for 0, 2, 4, 8, 16 weeks. Left panel, right of vertical solid line: plasma insulin levels of mice on a high fat diet for 17, 18, 19 or 20 weeks (HFD, filled symbols) and mice that were switched after 16 weeks of HFD-feeding to the LFD for 1, 2, or 4 weeks (LFD, open symbols). Right panel: plasma insulin levels of genetically obese *ob/ob* mice (filled symbols) with their littermate wild-type controls (open symbols) after 4 weeks of LFD feeding. Horizontal line indicates the mean for the plasma insulin levels.
(TIF)

S5 Fig Plasma glucose levels of mice. Left panel, left of vertical solid line: plasma glucose levels of mice on a low fat diet (LFD, open symbols) or high fat diet (HFD, filled symbols) for 0, 2, 4, 8, 16 weeks. Left panel, right of vertical solid line: plasma glucose levels of mice on a high fat diet for 17, 18, 19 or 20 weeks (HFD, filled symbols) and mice that were switched after 16 weeks of HFD-feeding to the LFD for 1, 2, or 4 weeks (LFD, open symbols). Right panel: plasma glucose levels of genetically obese *ob/ob* mice (filled symbols) with their littermate wild-type controls (open symbols) after 4 weeks of LFD feeding. Horizontal line indicates the mean for the plasma glucose levels.
(TIF)
