# Supplementary material for: Changes in Dietary Fat Content Rapidly Alters the Mouse Plasma Coagulation Profile without Affecting Relative Transcript Levels of Coagulation Factors
Source: PLoS One. 2015 Jul 15;10(7):e0131859. doi: 10.1371/journal.pone.0131859 (PMC4503443; doi:10.1371/journal.pone.0131859)
Supplement: S1 Table — (DOCX) [file pone.0131859.s001.docx]

**S1Table. Hepatic mRNA levels of coagulation genes of mice on a low fat diet (LFD) or high fat diet (HFD) for 16 weeks.**

|  | **LFD (n=10)** | **HFD (n=10)** |
| --- | --- | --- |
| Fibrinogen | 1 (0.86-1.17) | 1.02 (0.93-1.12) |
| Factor II | 1 (0.90-1.11) | 0.98 (0.95-1.01) |
| Factor VII | 1 (0.88-1.14) | 0.84 (0.76-0.92) |
| Factor VIII | 1 (0.87-1.15) | 0.81 (0.69-0.94) |
| Factor IX | 1 (0.90-1.11) | 1.00 (0.96-1.04) |
| Factor X | 1 (0.89-1.12) | 0.84 (0.82-0.86) |
| Factor XI | 1 (0.91-1.10) | 1.00 (0.93-1.08) |
| Factor XII | 1 (0.93-1.07) | 0.72 (0.69-0.76) ^‡^ |

Data are expressed as mean (minimum-maximum expression level). ^‡^p<0.001 as compared to LFD mice.
